# Point-of-care tests for syphilis and yaws in a low-income setting – A qualitative study of healthcare worker and patient experiences

**DOI:** 10.1371/journal.pntd.0006360

**Published:** 2018-04-19

**Authors:** Michael Marks, Tommy Esau, Rowena Asugeni, Relmah Harrington, Jason Diau, Hilary Toloka, James Asugeni, Eimhin Ansbro, Anthony W. Solomon, David Maclaren, Michelle Redman-Maclaren, David C. W. Mabey

**Affiliations:** 1 Clinical Research Department, Faculty of Infectious and Tropical Diseases, London School of Hygiene & Tropical Medicine, Keppel Street, London, United Kingdom; 2 Hospital for Tropical Diseases, University College London Hospitals NHS Trust, London, United Kingdom; 3 Atoifi Adventist Hospital, Atoifi, Malaita Province, Solomon Islands; 4 Department of Noncommunicable Disease Epidemiology, Faculty of Epidemiology and Public Health, London School of Hygiene & Tropical Medicine, London, United Kingdom; 5 College of Medicine and Dentistry, James Cook University, Cairns, Queensland, Australia; Centers for Disease Control and Prevention, UNITED STATES

## Abstract

**Introduction:**

The human treponematoses comprise venereal syphilis and the three non-venereal or endemic treponematoses yaws, bejel, and pinta. Serological assays remain the most common diagnostic method for all treponemal infections.

Point-of-care tests (POCTs) for syphilis and yaws allow testing without further development of infrastructure in populations where routine laboratory facilities are not available. Alongside the test’s performance characteristics assessed through diagnostic evaluation, it is important to consider broader issues when rolling out a POCT. Experience with malaria POCT roll-out in sub-Saharan Africa has demonstrated that both healthcare worker and patient beliefs may play a major role in shaping the real-world use of POCTs. We conducted a qualitative study evaluating healthcare worker and patient perceptions of using a syphilis/yaws POCT in clinics in the East Malaita region of Malaita province in the Solomon Islands. Prior to the study serology was only routinely available at the local district hospital.

**Methods:**

The POCT was deployed in the outpatient and ante-natal departments of a district hospital and four rural health clinics served by the hospital. Each site was provided with training and an SOP on the performance, interpretation and recording of results. Treatment for those testing positive was provided, in line with Solomon Islands Ministry of Health and Medical Services’ guidelines for syphilis and yaws respectively. Alongside the implementation of the POCT we facilitated semi-structured interviews with both nurses and patients to explore individuals’ experiences and beliefs in relation to use of the POCT.

**Results and discussion:**

Four main themes emerged in the interviews: 1) training and ease of performing the test; 2) time taken and ability to fit the test into a clinical workflow; 3) perceived reliability and trustworthiness of the test; and 4) level of the health care system the test was most usefully deployed. Many healthcare workers related their experience with the POCT to their experience using similar tests for malaria. Although the test was considered to take a relatively long time to perform the benefits of improved access to testing were considered positive by most healthcare workers. Qualitative data is needed to help inform better training packages to support the implementation of POCT in low-resource settings.

## Author summary

Syphilis and yaws are closely related bacterial infections. In many countries where the diseases are found there is limited access to diagnostic testing. Recently a point of care test for both diseases has been developed. In the current study we evaluated the experience of healthcare workers and patients in using the test in the Solomon Islands. Both healthcare workers and patients valued the improved access to testing that provided by the point of care test. Experience of healthcare workers in using similar tests for other diseases, such as malaria, had both positive and negative impacts on their beliefs about the syphilis and yaws test.

## Introduction

The human treponematoses comprise venereal syphilis and the three non-venereal or endemic treponematoses yaws, bejel, and pinta[1]. Syphilis, caused by *Treponema pallidum* subsp. *pallidum*, remains an important cause of both morbidity and mortality worldwide and remains one of the major preventable causes of stillbirth globally [2]. Stillbirths and neonatal death due to mother-to-child transmission of syphilis are almost entirely preventable through appropriate screening and treatment of pregnant women during antenatal care[3,4]; this intervention has been shown to be highly cost-effective [5], but access to syphilis testing is a barrier to implementation in many settings[6,7].

Yaws, caused by *T*. *p*. subsp. *pertenue* is the most common of the endemic treponematoses[1]. Although its aetiological agent is closely related to *T*. *p*. subsp. *pallidum*, yaws is transmitted by non-sexual skin to skin contact affects children living in poor, rural communities in the tropics, where the ambient humidity is high[8]. In 2012, the World Health Organization (WHO) launched a renewed plan to eradicate yaws globally by 2020 using community mass treatment with single dose azithromycin. Currently, when submitting data to WHO, most countries report clinically suspected cases without laboratory confirmation, but clinical diagnosis may be inaccurate, with less than 50% of phenotypically consistent cases confirmed serologically in some studies [9,10]. The development and validation of appropriate point-of-care diagnostics for use in yaws eradication efforts has been highlighted as a research priority[11], and would allow strengthening of national yaws surveillance programmes and accurate reporting of cases.

Serological assays remain the most common diagnostic method for all treponemal infections. Importantly, none of the currently available assays is able to differentiate between infection with syphilis and infection with any of the endemic treponematoses[12]. Standard serological testing consists of both a treponemal specific test, such as the *Treponema pallidum* particle agglutination assay (TPPA), combined with a non-treponemal test, such as the Rapid Plasma Reagin (RPR) assay. Treponemal tests are highly specific but generally remain positive for life following infection. Non-treponemal tests are less specific but reflect disease activity more accurately, and their titres fall following successful treatment. Testing therefore requires both assays to give an interpretable result.

Whilst traditional serological tests are relatively straightforward to perform, they require a cold chain and electricity, denying access to testing to those living in many remote communities. Point-of-care tests (POCTs) allow testing without further development of infrastructure in populations where routine laboratory facilities are not available. A large number of syphilis POCTs meeting the ASSURED criteria[13] have been developed [6] but the majority of commercially available tests include only a treponemal line. Although these tests allow identification of individuals who have been exposed to treponemal infection, they cannot distinguish between current and previous, successfully treated infection. In the antenatal setting, this results in the unnecessary treatment of women with previously treated syphilis; in yaws eradication programmes it represents a barrier to accurately assessing ongoing transmission following community mass treatment.

A single commercially available test, the Dual Path Platform (DPP-POCT) Syphilis Screen and Confirm test kit (Chembio, Medford, NY, USA) provides both a “treponemal” result (analogous to a TPPA assay) and a “non-treponemal” result (analogous to a qualitative RPR assay)[14], and can therefore distinguish between current and past infection. A number of studies and a meta-analysis have demonstrated that the test has a good sensitivity and specificity for the diagnosis but there have not been any evaluations of implementation of the test in a real world setting[15–19].

Alongside the test’s performance characteristics assessed through diagnostic evaluation, it is important to consider broader issues when rolling out a POCT. Adequate training and quality control (QC) steps must be developed, and both health-care workers and patients may require additional education about the role of POCTs in making a diagnosis. Experience with malaria POCT roll-out in sub-Saharan Africa has demonstrated that both healthcare worker and patient beliefs significantly influence the utilisation and interpretation of POCTs and may play a major role in shaping their use in real-world settings[20,21].

The Solomon Islands reports the third most cases of yaws in the world annually[8], after Ghana and Papua New Guinea. Recent ANC surveys have also show a high prevalence of syphilis[22]. Diagnostic testing in the Solomon Islands is limited to hospital laboratories. As a result testing for syphilis in rural clinics relies on sending tests away to a local hospital, whilst most cases of yaws are never confirmed serologically. These gaps in current diagnostic test provision highlight a potential role for the DPP-POCT in the Solomon Islands to improve access to testing for both yaws and syphilis. We conducted a qualitative study evaluating healthcare worker and patient perceptions of using a syphilis/yaws POCT in clinics in the East Malaita region of Malaita province in the Solomon Islands.

## Methods

### Study site

The study was at Atoifi Adventist Hospital (AAH), Uru Harbour, the local referral hospital for the eastern region of Malaita Province. Addition healthcare services are provided in the Province by a number of nurse aid posts and rural health clinics. Serological testing for syphilis and yaws is available in this catchment area but requires venepuncture and delivery of the blood sample to AAH or for the patient to travel to the hospital for venepuncture. In many remote communities this may require travelling for more than eight hours to have blood taken.

### The DPP-POCT

DPP-POCT kits were purchased from Chembio for use in the study. The DPP-POCT combines both a treponemal and a non-treponemal line. A finger prick blood sample is collected and placed with buffer into the first well on the POCT. The test is allowed to run for five minutes before additional buffer is added to the second well on the POCT. The test is allowed to run for a further 10–15 minutes before the results are read. Tests were purchased by the London School of Hygiene & Tropical Medicine. Test kits and equipment required to perform the DPP-POCT were provided to all clinics for the duration of the study.

### Introduction of the DPP-POCT

The DPP-POCT was deployed in three settings: the outpatient department of AAH, the antenatal clinic of AAH and four rural health clinics accessible to AAH only by walking or canoe. Clinical care in each of these settings is provided by registered nurses. The rural clinics all provide antenatal care as well as general outpatient care including seeing patients with yaws. Members of the study team visited each location and provided a one-day training package to staff members on the use of the DPP-POCT. Each location was also provided with a standardised protocol for performance, interpretation and recording of results. Briefly this protocol included collection of a finger-prick blood samples followed by performance and interpretation of the assay in line with manufacturer’s instructions. Treatment for those testing positive was provided, in line with Solomon Islands Ministry of Health and Medical Services’ guidelines for syphilis and yaws respectively.

### Interviews

We facilitated semi-structured interviews with both health care workers and patients to explore individuals’ experiences and beliefs in relation to the DPP-POCT. Participants were selected using convenience sampling of health-care workers at each facility who reported having used the DPP-POCT. The interviews covered experience of training, positive and negative experiences of performing the test, perceptions of reliability of the test and what level of the healthcare system that individuals believed the test should be available at. For health care workers interviews were performed by researchers with training and previous experience in qualitative research in the Solomon Islands. We performed interviews at each clinical location one and six months after the rollout of the POCT aiming to interview each healthcare worker in each clinical settings. At six months, we used convenience sampling to additionally interview patients attending clinics about their experience of being tested using the POCT. All interviews were undertaken in either Kwaio (a local language) or Pijin (the lingua-franca of the Solomon Islands). Interviews were digitally recorded and then transcribed for analysis by a member of the study team (TE).

### Sample size

As this was a qualitative study, no formal sample size calculation was undertaken. In each setting purposive sampling of health workers was used to select participants. In each clinical setting only one cadre of staff was available, either nurses in rural clinics and the AAH outpatient department and midwives in the AAH ANC department. We aimed to perform multiple interviews at each clinic and at each time point to ensure a full range of views and experience of the DPP-POCT were obtained until we reached saturation.

### Analysis

Immersion in the data was achieved by verbatim transcribing of the interviews and repeated reading of interview transcripts prior to their analysis. We used thematic analysis to identify common themes or stands in healthcare worker and patient experiences.

### Ethics

The study was approved by the ethics committees of the Atoifi Adventist Hospital, the Solomon Islands Ministry of Health and Medical Services, and the London School of Hygiene & Tropical Medicine. Individuals undergoing testing with the DPP-POCT and individuals participating in interviews provided written informed consent. Where a DPP-POCT was performed on a child we obtained written consent from their parent or guardian and additionally obtained verbal assent from the child.

## Results

Over the study period approximately 500 DPP-POCTs were used across the study sites. We interviewed a total of 20 health care workers, 12 at the one month time point post training and a further eight healthcare workers at the six month time point post training. We additionally interviewed four patients who had been tested using the DPP-POCT.

### Themes

As a result of the thematic analysis we identified four main themes: 1) training and ease of performing the test; 2) time taken and ability to fit the test into a clinical workflow; 3) perceived reliability and trustworthiness of the test; and 4) level of the health care system the test was most usefully deployed.

### Training and ease of testing

All but two healthcare workers interviewed (18/20) had received training in the use of the DPP-POCT from members of the study team at the time of the DPP-POCT pilot. All healthcare workers reported that they were already familiar with POCTs for malaria and that this helped them conduct the DPP-POCT;; *“bikos hemi similar wetim RDT blo malaria (because the test is similar to the RDT for Malaria)(*Healthcare worker, rural health clinic*)”*. The majority of healthcare workers reported that the test was relatively easy to perform but several noted that it was possible to make mistakes with both the timing and volume of buffer resulting in errors in the test

*“hia test kit ia bae hem onle problem*, *if mi makem mistake lo namba of pufs mi duim mistake lo hem*, *or taeming blo mi hem no accurate hem nao bae mi save westim kit blo mi yia (the only problem is if you make a mistake with the number of drops or the timing the test isn’t accurate and is wasted) (*Healthcare worker, rural health clinic*)”*

One healthcare worker explained the withdrawal of the blood for the test was sometimes difficult; “*wan nogud samting lo saed lo staff nomoa, lo saet lo withdrawal lo blud. Hem nomoa hem lelbet danger fo saed lo staff” (one negative thing for staff was taking blood. That was a bit of a danger for the staff* (Healthcare worker, rural clinic).

### Time for testing

Several nurses (4/20) reported that the DPP-POCT took a relatively long time to deliver a result and that this could disrupt workflow in a clinical environment. One health care worker explained,

*“Lelebet part hemi nogud nomoa lelebet taeming wea iu tekem fo redim resolt; wea hem rekwaerim 20–25 minutes fo iu redim resolt yia*. *(A thing that is not good is the time it takes to read the result; it can require 20 minutes before you can read the result)* (Healthcare worker, rural health clinic)*”*

Despite this delay, healthcare workers at both AAH and the clinics reported that the time taken for the test compared favourably with having to wait a week for a result to come back from the hospital laboratory, or the time it would take patients to reach facilities where standard laboratory tests could be performed. A health care worker from AAH stated that previously,

*“last taem ia*, *bae mifala givim go lo lab afta wan wik na bifo mifala resivim resolt (before when we have the sample to the lab we had to wait a week to receive the result)”* (Healthcare worker, Atoifi Hospital Antenatal Clinic)*”*.

A rural healthcare worker explained

*“15 minutes ia hem sort taem ia compared lo oketa test before wea laek lo siteton lo hia samfala lo oketa like antenatal mothers oketa hev to walk fo 3*,*4*,*5 hours ia fo casim facilitI (15 minutes is a short time compared to other tests where the antenantal mothers might have to walk 3*,*4*,*5 hours to reach a facility)* (Healthcare worker, rural health clinic)*”*

### Perceived reliability of results

Most nurses (16/20) reported confidence in the results of the DPP-POCT and that this had been reinforced by concordance between POCT and laboratory tests. Two nurses highlighted the experience of discordant results between laboratory and POCT testing as impacting their confidence in the test. One healthcare worker stated,

*“wea oketa test fo syphilis so taem diswan hem confirm positive and mefala sendim blud ah same resolt hem araef*, *so that’s how mi garem trust det diswan ah hem confirm test*. *(We always test for syphilis*. *When the test is positive and I send the blood away and get the same result from the lab that gives me trust in the test* (Healthcare worker, rural health clinic)*”*

Another healthcare worker at a different clinic explained, *“wanfala patient na taem mefa checkim lo RDT hem negativ but den mefala cross check lo down hem reactive na blud blo hem so hem na mifala stat kwestion about diss test (One patient was negative on the RDT but reactive on the blood which makes me question the test)* (Healthcare worker, rural health clinic)*”*

Parallels were also drawn between the experience of RDTs for malaria and the DPP-POCT. Two nurses invoked previous experience of discordant results between the POCT used for malaria and microscopic diagnosis of malaria, and reported that this made them worry, by analogy, if the DPP-POCT was accurate.

*“Lo malaria kit if malaria wea hem 1 plus disfala RDT blo malaria yia hemi no readim yia*..*Bae olwes givim mifala negatif ansa*.*But sapos mi givim go lo microscopist bae hemi say 1 plus*. *So det wan ia nao hemi givim mi daot lo disfala syphilis becos hemi no givim mi how seriousness nao disfala diseses*. *(If the slide has 1+ PF then the malaria RDT is not detecting it*. *The test is always negative; but if you give it to the microscopist they report 1+*. *So that gives me doubts about the syphilis test*, *because it is a serious disease)* (Healthcare worker, rural health clinic)*”*

### Access to testing

All health care workers (20/20) reported that providing access to testing at the clinic level was a strength of the POCT. Lack of access to laboratory facilities due to the distance from the hospital was seen as a significant challenge in terms of logistics, time and costs. POCT addressed these challenges by providing access to testing at a lower level of the health care system. One nurse stated *“bikos lab hem farawe from mifala*. *So hem barava best ples na ia*. *(The lab is far away from us*. *So here (clinic) is the best place)* (Healthcare worker, rural health clinic*)”*. Another rural health worker explained,

*“Hem nao ekspensif fo olketa mama fo cum daon lo hospital fo usem test ia*. *Iumi save duim noma lo olketa rural clinics (It is expensive for the mothers to go to the hospital (AAH) to have the test*. *Lets do the test at all of the rural clinics* (Healthcare worker nurse, rural health clinic).

Patients reported that improving access to testing at the clinic level was beneficial due to the delays involved in travelling to hospitals for testing. One patient explained,

*“sendem go lo hospital o sendem go lo olketa nara place testim kam olketa blud ia ating bae hem lelebet slo to so ating hem gud na for okelta makem lo hia nomoa (being sent to a hospital or another place for blood tests is slow so it is good to be able to take it here)* (Patient rural health clinic).

The costs of getting to the hospital was also a concern expressed by for health workers.

## Discussion

In this study we provide the first real-world evaluation of the acceptability of the DPP-POCT in routine health care settings, in a country co-endemic for yaws and syphilis. Whilst a number of evaluations have been conducted of the analytic performance of the DPP-POCT[14,18,19], no previous studies have addressed factors affecting its real-world roll-out. Our data highlight the ability of these tests to improve access to diagnostics for patients in remote communities, and the receptiveness to the test of healthcare workers and patients. Our findings also highlight lessons that can be learnt in guiding roll-out of the DPP-POCT to support both yaws and syphilis control programmes.

The majority of nurses reported a high level of trust in the test, and both patients and nurses recognised the benefits of making diagnostic testing available in primary health facilities without recourse to a hospital laboratory. The test was generally reported to be easy to perform based on the training received in the study and despite the relatively long time required to obtain a result, nurses reported the test could be integrated into clinical workflows. Previous studies of the roll-out of rapid syphilis tests in other settings have also demonstrated an overall high level of acceptability and feasibility [23]. In line with our findings, these studies highlighted the potential value of reducing travel time to access tests and the ability to offer same day treatment and testing to patients.

In the current study, many health care workers drew parallels between their experience of the DPP-POCT and their previous experience with malaria RDTs. Whilst familiarity with RDTs was clearly advantageous, it also highlighted how lack of confidence in one test kit could affect the roll-out of a second, unrelated, test kit. Adoption of RDTs for the diagnosis of malaria has not always translated into anticipated reductions in the number of patients treated for malaria in the real world[24]. Studies of RDT roll-out programmes have highlighted the complex interplay between patient and clinician expectations that drives how clinicians interact with, utilise and interpret RDTs[25]. Taking account of these interactions and building more sophisticated training and support packages are important facilitators for successful adoption of RDTs[21]. Our data might inform a refined DPP-POCT training package.

There are a number of limitations to this study. Firstly, we focused on a relatively small number of clinics over a fairly limited timeframe. This reduced our ability to consider the impact of broader health systems factors that may influence the feasibility of rolling out the DPP-POCT. Previous studies on syphilis RDTs have highlighted the importance of supply chain and quality control monitoring systems in maintaining long term effectiveness of RDT scale-up at a national level[23]. These cross-cutting issues would be important to consider in the context of a broader programmatic scale up of the DPP-POCT. Second, we did not consider issues around the cost of introducing the test kit, either from the perspective of eliminating mother-to-child-transmission of syphilis or from the perspective of supporting yaws eradication. Whilst antenatal screening for syphilis is considered a highly cost-effective intervention[5], the optimal strategy depends on the prevalence of syphilis and the cost of the test kits. In many situations, use of a treponemal-only rapid syphilis test is more cost effective, albeit at the cost of over-treatment[5]. Cost-effectiveness studies on the use of RDTs for yaws have advocated that a two stage screening process, an initial treponemal-only test followed by a DPP-POCT if positive, is more cost-effective, given the current cost of the DPP-POCT (Fitzpatrick REF–accepted PLOS NTDs).

This is the first study to assess the acceptability of rolling out the DPP-POCT in a routine health care setting. We highlight the potential added value to both patient and healthcare workers that can be provided by positioning these tests at the primary health care level. Longer term and larger evaluations of DPP-POCT would be valuable to assess the impact and cost-effectiveness of scaling up access to the DPP-POCT on the management of syphilis and yaws.

## Supporting information

S1 Checklist STROBE checklist. (DOCX)
